# Case Report: Corneal Inlay Removal after Myofibroblast Detection under *in Vivo* Confocal Microscopy

**DOI:** 10.1097/OPX.0000000000002018

**Published:** 2023-04-13

**Authors:** Suzu Yoshitomi, Taiichiro Chikama, Yoshiaki Kiuchi

**Affiliations:** 1 Department of Ophthalmology and Visual Science, Graduate School of Biomedical and Health Sciences, Hiroshima University, Hiroshima, Japan; ∗ chikama@hiroshima-u.ac.jp

## Abstract

Corneal inlays, one of the treatment options for presbyopia, are effective and safe, and various such devices have been developed. However, there have been cases of inlay removal due to complications or patient dissatisfaction.

**PURPOSE:**

The purpose of this study was to report a case of inlay removal due to corneal opacity after inlay implantation and the results of 5 years of follow-up.

**CASE REPORT:**

A 63-year-old man was referred to our hospital with visual disturbance and double vision in his left eye. Two years before presentation at our hospital, he had undergone bilateral laser *in situ* keratomileusis with corneal inlay implantation in the left eye at another clinic. Slit-lamp examinations showed paracentral corneal opacity. The patient was treated with tranilast eye drops for 18 months, with no progression of symptoms. However, 6 months after stopping the eye drop treatment, the opacity recurred, and vision acuity decreased, along with the formation of myofibroblasts around the inlay, as revealed by *in vivo* confocal microscopy. Consequently, the inlay was removed at the previous clinic. During the subsequent 5-year follow-up period, ophthalmic examination revealed reduced corneal opacity, although visual acuity did not change; moreover, no myofibroblast was found.

**CONCLUSIONS:**

Corneal inlays can sometimes cause complications. In this case, the patient experienced corneal fibrosis and associated vision loss. *In vivo* confocal microscopy detected myofibroblasts that cause corneal stromal fibrosis; thus, the removal was decided to avoid fibrosis progression.

**Figure:**
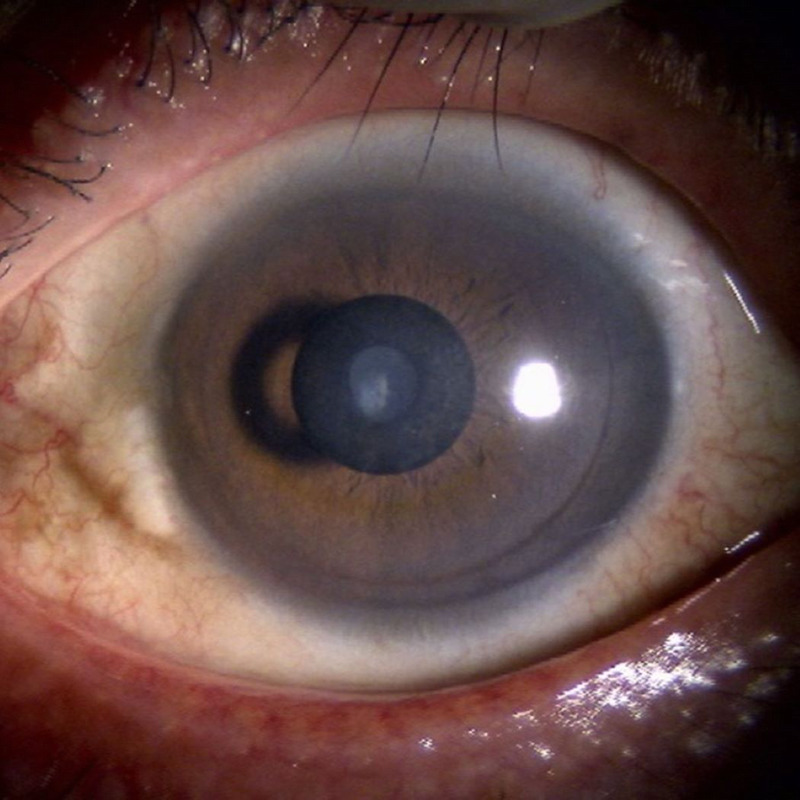


A healthy cornea exhibits transparent cellular structure and the ability to refract or focus incoming light. These properties are responsible for vision and are indispensable to the quality of vision. Advances in refractive surgical techniques have resulted in improved vision among young people, enabling them to discard their old eyeglasses or contact lenses. However, near vision deterioration due to age-related presbyopia often results in the need for eyeglasses once again. Various treatments of presbyopia have been developed, for example, the multifocal intraocular lens that can improve near vision by adjusting the refractive index of the lens, the small-aperture intraocular lens by extending the depth of focus, and the accommodating lens by changing the refractive power of the eye through transmitting ciliary muscular contractions.

Other presbyopia treatments target the cornea, for example, laser-assisted *in situ* keratomileusis (LASIK), conductive keratoplasty, and corneal inlays. Several corneal inlays, such as the KAMRA inlay (AcuFocus, Inc., Irvine, CA), have been developed to improve near and intermediate vision for patients with presbyopia. The KAMRA inlay is designed to increase the depth of focus by a pinhole effect.^1^ The current product is made of polyvinylidene fluoride with a 5-μm thickness, a 3.8-mm diameter, and a 1.6-mm central aperture with 8400 micropores to facilitate the flow of oxygen or nutrition through the cornea. Improved products have so far been reported safe and effective, although complications have been reported with older types because of their thickness (10 μm), fewer holes (1600 holes), and lower oxygen transmissibility.^1–4^ However, some patients implanted with inlay may suffer from loss of contrast sensitivity, prolonged vision recovery after LASIK, and dry eyes, depending on the depth of corneal inlay.^2,3,5^

Fibrosis is the excessive accumulation of extracellular matrix components such as collagen and fibronectin, and it can affect any organ and may sometimes be fatal.^6^ When fibrosis occurs in the cornea, the structural changes often induce corneal astigmatism and reduced transparency, resulting in visual disturbance.

We present a case of corneal fibrosis after the implantation of a KAMRA inlay for refractive treatment, resulting in vision loss. Detecting myofibroblasts using *in vivo* confocal microscopy aided the decision to proceed with surgical treatment. Removing the inlay caused the structural protrusion and myofibroblasts' disappearance, but the stromal opacity persisted during long-term observations.

## CASE PRESENTATION

**FIGURE 1:**
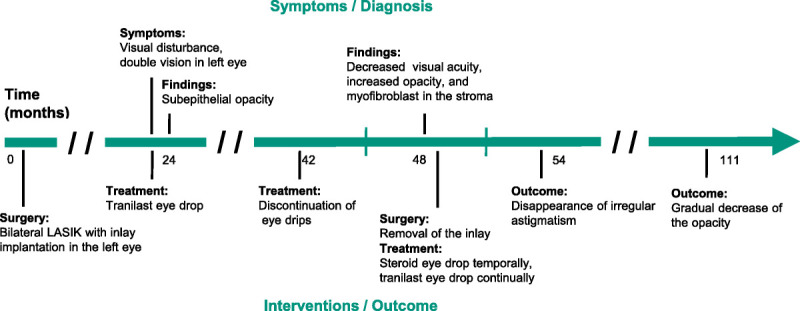
Clinical timeline: 63-year-old man had surgical and eye drop treatment of complication of corneal inlay. LASIK = laser *in situ* keratomileusis.

A 63-year-old man was referred to our hospital with visual disturbance, double vision in his left eye, and a feeling of discomfort under dim light. Two years prior, this patient had undergone bilateral LASIK with KAMRA inlay implantation in the left eye (Fig. 1).

**FIGURE 2:**
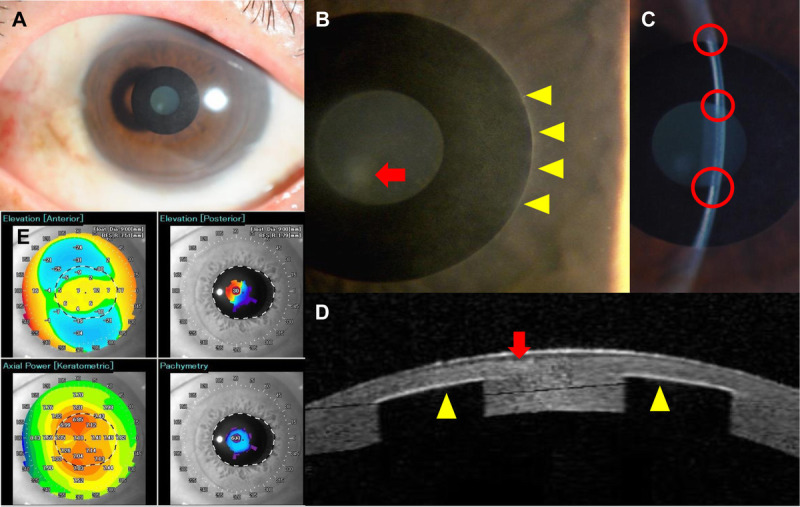
Slit-lamp microscopic and AS-OCT findings at the initial visit. (A) There was no injection and inflammatory cell in the anterior chamber of the left eye. Slit-lamp microscopy showed opacity at the paracentral cornea, inside the inlay (B, red arrow), iron line along with the inlay outline (B, yellow arrowhead), and a gap at the flap edge (C). (D) Anterior-segment optical coherence tomography revealed subepithelial opacity inside the inlay (red arrow) and increased reflection along with the inlay (yellow arrowhead). (E) Keratometry showed astigmatism. AS-OCT = anterior-segment optical coherence tomography.

On his first visit to our hospital, the patient's uncorrected distance visual acuities in the right and left eyes were 20/12.5 and 20/20, respectively. Intraocular pressure measurements in the right and left eyes were 17 and 18 mmHg, respectively. Slit-lamp examination revealed increased reflection along the rim of the inlay, subepithelial opacity inside the inlay, and iron lines along the contour of the inlay. Anterior-segment optical coherence tomography (CASIA SS-1000 OCT; Tomey, Nagoya, Japan) revealed hyperreflection around the inlay and thickened central epithelium (Fig. 2), and the depth of the inlay was 235 μm of the 626-μm corneal central thickness. The patient was treated with tranilast eye drops (RIZABEN Eye Drops 0.5%; Kissei Pharmaceutical Co., Ltd., Tokyo, Japan) three times daily for the opacity. During 18 months of this treatment, we observed no further progression in corneal opacity, and the tranilast treatment was stopped temporarily.

**FIGURE 3:**
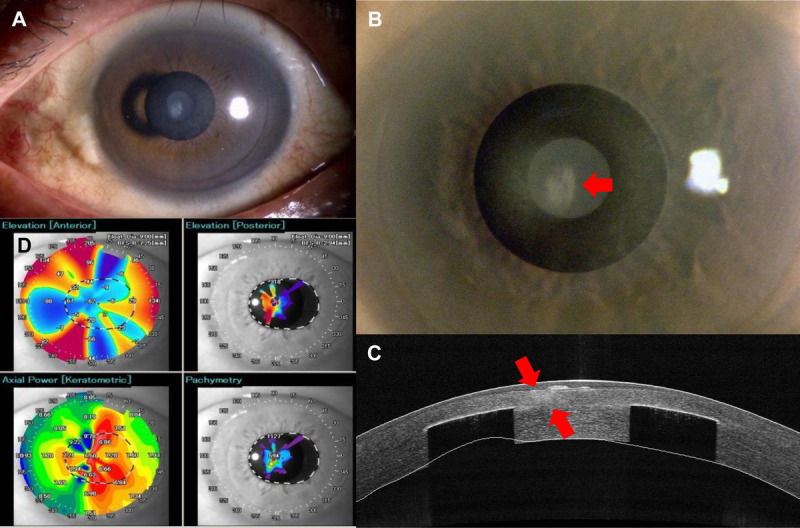
Slit-lamp microscopy and AS-OCT images before the removal of the inlay. (A) Slight conjunctival injection was observed, but there was no sign of infection. (B) Paracentral opacity had increased in both size and intensity compared with the condition of the cornea 2 years prior (red arrow). (C) Anterior-segment optical coherence tomography revealed the opacity extended from the stroma beside the inlay and at the depth of the inlay to the shallow region of the stroma and subbasal area (red arrow). (D) Keratometry showed irregular astigmatism. AS-OCT = anterior-segment optical coherence tomography.

**FIGURE 4:**
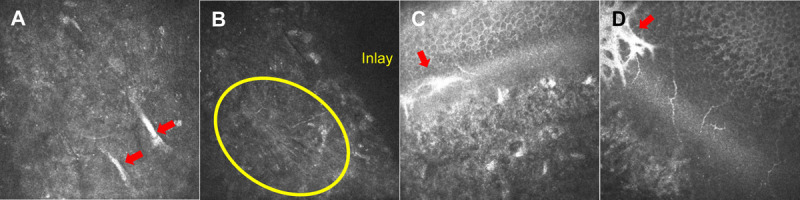
*In vivo* confocal microscopy before inlay removal. (A) Myofibroblasts were observed at the same depth as the inlay (red arrow). (B) Fibrotic tissue was produced by myofibroblasts located beside the inlay (inside yellow circle). (C and D) Sagittal sections of the opaque area, showing the fibrosis reaching the subbasal area of the Bowman's layer (red arrow).

Six months after stopping tranilast treatment, the uncorrected distance visual acuity value in the left eye decreased from 20/20 to 20/32. Slit-lamp evaluation revealed that the round, white opacity had increased from a paracentral location to the basement membrane, resulting in irregular astigmatism (Fig. 3). In addition, *in vivo* confocal microscopy revealed the presence of myofibroblasts with fibrotic changes in the subbasal area and the shallow stroma (Fig. 4). Based on these findings, the patient decided to have the corneal inlay removed at the clinic that performed the original operation. Post-operative therapy consisted of betamethasone eye drops three times daily for 2 months and subsequent fluorometholone eye drops three times daily for the next 9 months. In addition, tranilast eye drops were used four times daily for 2 years, tapering to once a day, which continued until the last visit.

**FIGURE 5:**
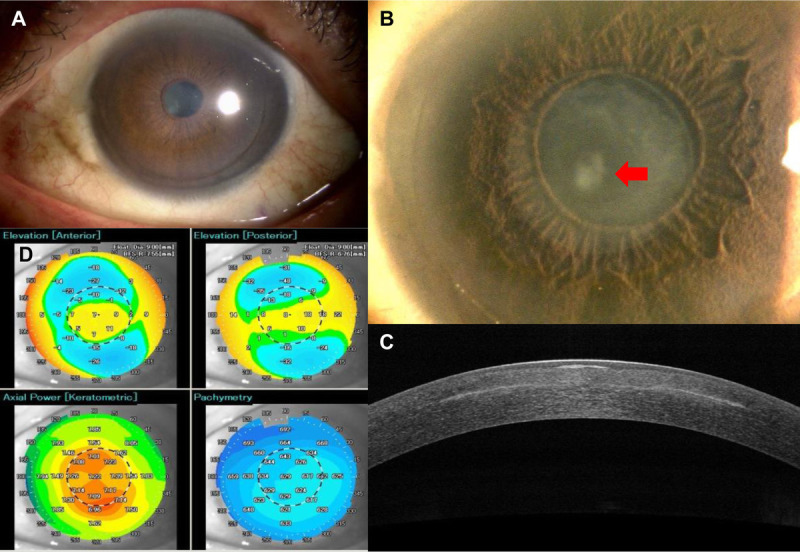
Slit-lamp microscopy and AS-OCT images at 6 months after the removal of the inlay. (A) After removing the inlay, there was no sign of infection. (B) Slit-lamp evaluation showing that the size of the corneal opacity had changed very little compared with the opacity before inlay removal (red arrow). Reduction in the size of the range of hyperreflection in AS-OCT (C) was observed along with disappearance of irregular astigmatism (D). AS-OCT = anterior-segment optical coherence tomography.

At 6 months after the explantation, the uncorrected distance visual acuity of the left eye remained at 20/32. However, slit-lamp examination showed a slight decrease in opacity in the corneal stroma (Fig. 5). This decrease in opacity gradually continued throughout a 5-year follow-up period. However, the visual acuity of the left eye remained unchanged (uncorrected distance visual acuity, 20/32). Clinical symptoms and complaints of dry eye were not observed during the follow-up period. Periodic *in vivo* confocal microscopy revealed no myofibroblasts.

## DISCUSSION AND CONCLUSIONS

This report documents the clinical course of a case of corneal fibrosis with opacity after KAMRA inlay implantation and the long-term outcome after its removal. Corneal opacity was first observed after the patient complained of a haze in his eye 2 years after inlay surgery. The use of *in vivo* confocal microscopy allowed the detection myofibroblasts, which led to the decision of inlay removal. Therefore, eye care providers need to observe and control the eyes of patients with KAMRA inlay implants for a long period. In some cases, it may be necessary to extract the inlay to prevent the progression of complications.

The safety and effectiveness of the corneal inlay have been established in several reports. In addition, it can be easily removed when needed.^7^ Indeed, 1.8 to 10.3%^2,8–10^ of corneal inlay implants have been subsequently removed up to 36 months post-operatively. However, none of these cases have had a significant visual loss before and after inlay removal. The operation is considered uneventful and with no post-operative vision loss as long as the inlay is removed within 1 year post-operatively.^2,4,8^ The primary reasons for removal in these cases include visual complaints such as refractive changes or not meeting patients' expectations. Moreover, the shallower depth of inlay implantation is associated with removal due to visual complaints.

In one case, visual loss occurred after an extended follow-up period. Romito et al.^11^ reported a case of persistent corneal fibrosis after the explantation of a KAMRA inlay that had been in place for 6 years. In this report, the patient's corrected distance visual acuity decreased significantly from 20/20 to 20/200 in 6 years. In addition, stromal fibrosis occurred at the inlay site. Eight months after the explantation of the inlay, the corrected distance visual acuity improved only slightly (20/100), whereas the patient's hyperopic shift persisted. Probable causes of corneal fibrosis include the inhibition of oxygen and glucose diffusion and chronic immunological reaction due to the inlay.^11^

The depth of inlay implantation in our case was shallower than 40% of the total thickness, and the patient's visual acuity decreased gradually. *In vivo* confocal microscopy revealed some myofibroblasts located beside the inlay before removal that were not identified in the previous report.^11^ Such myofibroblasts often play an essential role in wound healing responses to trauma, surgery, and infections. However, excessive production of myofibroblasts causes vision-reducing fibrotic scars.^12^ Here, the structural changes associated with fibrosis caused protrusions on the corneal surface, resulting in irregular astigmatism, and the patient complained of a “visual disturbance.” During the early phase of fibrosis formation, we observed no astigmatism affecting visual acuity, and the hyperreflective area seen on anterior-segment optical coherence tomography images extended only to the area beside the inlay. As the fibrosis gradually progressed, anterior-segment optical coherence tomography revealed vertical progress of the opacity, along with irregular astigmatism.

After removing the inlay, fibrosis progression stopped, as seen via slit-lamp microscopy and anterior-segment optical coherence tomography. In addition, *in vivo* confocal microscopy showed no myofibroblasts. The progression or decrease of fibrosis depends on the balance between the processes of mitosis and apoptosis of myofibroblasts.^12^ Once the myofibroblasts in the fibrotic area are eliminated, they are replaced by keratocytes, which then remove disordered extracellular matrix and gradually reestablish the collagen fibers associated with stromal transparency.^13,14^ However, we observed no keratocyte activation as myofibroblast production decreased after removing the inlay. This result suggests that the inlay was responsible for continuously stimulating keratocyte transformation into myofibroblasts.

In this case, fibrosis developed, and his visual acuity decreased after tranilast treatment was stopped. Tranilast suppresses the release of transforming growth factor β, of which the stimulation activates fibroblasts into myofibroblasts.^15^ Originally used as an antiallergic drug,^16^ oral administration of tranilast is an adjuvant therapy for keloids and hypertrophic scars of the skin given its inhibitory effect on collagen synthesis in skin fibroblasts.^17^ In ophthalmic diseases, it is also expected to be used as a treatment of conditions other than allergic conjunctivitis given its inhibitory effects on fibrovascular proliferation and collagen deposition.^18^ Furthermore, tranilast has been used effectively in the treatment of corneal keloids,^19^ which originate from fibrovascular tissue^20^ or within the stroma of the cornea. Therefore, despite the limitation of one case, all pieces of evidence suggest that tranilast suppresses the activation of keratocytes, which leads to fibrosis. This provides an explanation of the way tranilast affected the patient's fibrosis in this case. However, further studies are required to test this theory.

In conclusion, we experienced a case of fibrosis with visual loss after inlay insertion. The detection of myofibroblasts by *in vivo* confocal microscopy led to the decision to remove the inlay. Findings of *in vivo* confocal microscopy can be very effective in determining a therapeutic strategy.
